# Dynamic pathways in the development of university students’ AI literacy: integrating quantitative and qualitative evidence

**DOI:** 10.3389/fpsyg.2026.1822027

**Published:** 2026-06-04

**Authors:** ZhiHui ZhuGe, Chengshi Li

**Affiliations:** 1 School of Marxism, Taishan University, Tai’an, China; 2 Department of Education, Gachon University, Seongnam, Republic of Korea

**Keywords:** artificial intelligence, artificial intelligence capabilities, high education, influencing factors, literacy

## Abstract

**Introduction:**

This study investigates the formative mechanisms underlying university students’ artificial intelligence (AI) literacy, focusing on the complex interrelationships among external support, AI self-efficacy, AI anxiety, and AI literacy.

**Methods:**

An integrated framework was developed and tested using a mixed-methods approach, combining structural equation modeling (SEM) with qualitative interviews of 15 high-anxiety, high-literacy (HAHL) students.

**Results:**

SEM results indicated that external support positively predicted AI literacy (*β* = 0.163, *p* < 0.001) and AI self-efficacy (*β* = 0.454, *p* < 0.001), while also increasing AI anxiety (*β* = 0.505, *p* < 0.001). Both AI self-efficacy (*β* = 0.728, *p* < 0.001) and AI anxiety (*β* = 0.527, *p* < 0.001) significantly and positively influenced AI literacy, highlighting that moderate anxiety may serve as vigilance-driven motivation to foster learning. Qualitative analyses further revealed that HAHL students translate anxiety into structured engagement through cognitive appraisal, resource accumulation, and professional identity reflection.

**Conclusion:**

These results demonstrate the dynamic interplay of environmental, cognitive, and affective factors in shaping AI literacy, and show how qualitative insights complement quantitative SEM analysis by revealing mechanisms behind anxiety-driven engagement. The study provides evidence-based guidance for higher education institutions to enhance AI literacy through optimized support systems, fostering technological confidence, and strategically managing AI-related anxiety.

## 1 Introduction

Contemporary society is undergoing profound transformation driven by artificial intelligence technologies (Kumar et al., 2024). From the rise of generative AI to the widespread adoption of intelligent decision-making systems, artificial intelligence is no longer a distant science-fiction concept but has become a fundamental force permeating education, industry, and social governance (Zhang et al., 2025). Against this backdrop, Artificial intelligence (AI) literacy has emerged as an essential competency for citizens in the information era (Shiri, 2024). This is particularly crucial for university students, who will soon participate in social and economic development and therefore must possess the core abilities and key competencies required in an AI-mediated society (Toker Gokce et al., 2025). AI literacy extends beyond traditional information technology skills; it refers to an individual’s comprehensive capacity to effectively and responsibly acquire, understand, evaluate, create, and communicate information in highly digitalized and intelligent environments, as well as to use AI tools and technologies for learning, innovation, problem-solving, and social participation (Lee et al., 2024).

Enhancing university students’ AI literacy carries both urgent practical significance and long-term strategic value. From the perspective of individual development, AI literacy is a foundational competency that enables students to remain competitive in the rapidly evolving talent landscape. Students with higher levels of AI literacy are more likely to demonstrate stronger adaptive capacity, problem-solving efficacy, and lifelong learning potential. Conversely, insufficient AI literacy may deepen the “digital divide” at the individual level—students may fail to leverage cutting-edge tools to optimize their learning, may be marginalized in collaborative tasks, and may face the risk of being replaced by automated processes or misaligned with the requirements of intelligent workplaces. However, in stark contrast to this urgent need is the current uneven and insufficient development of AI literacy among university students. Although many universities have actively responded by offering general education courses on AI, the effectiveness of such initiatives varies considerably.

AI education has increasingly become a key priority within national education systems (Prasetya et al., 2025). The Republic of Korea has established a relatively coherent and structured framework for informatization and AI education (Lee et al., 2022). AI and digital education have been integrated into formal curricula, accompanied by reforms in teacher training and instructional design, reflecting the Republic of Korea’s aim to cultivate future-ready talent through comprehensive AI-driven educational reform (Kim et al., 2024). In recent years, AI literacy has gradually become a prominent topic in educational research, with studies focusing on its conceptual foundation, core components, measurement tools, and educational practices. Overall, the development of AI literacy research shows a clear trend—from constructing conceptual frameworks to implementing curricula across educational stages and developing assessment tools. First, regarding conceptual foundations, several studies have attempted to redefine essential learning competencies in the AI era from an interdisciplinary perspective. AI literacy should encompass not only an understanding of technological principles but also the ability to interact with AI systems, evaluate them critically, and make ethical judgments concerning potential risks (Laupichler et al., 2022). Within the AI literacy framework, researchers emphasize four core dimensions: knowledge, skills, attitudes, and ethical awareness (Chiu et al., 2024). As AI literacy theories have matured, scholars have increasingly focused on practical pathways across educational stages. In early childhood education, young learners face challenges in grasping abstract AI concepts, yet approaches such as game-based learning and robot programming have proven feasible for introducing AI literacy to children (Su et al., 2023). At the secondary education level, research has centered more on project-based learning and interdisciplinary integration. For example, studies evaluating project-based AI literacy curricula in secondary schools found that such curricula significantly enhance students’ understanding of AI mechanisms and improve their problem-solving abilities (Kim and Lee, 2022). Similarly, research in technology education emphasizes that AI literacy is not merely technical knowledge but must integrate engineering, ethics, and social understanding (Stolpe and Hallström, 2024). Empirical studies on AI literacy courses in higher education have also shown that systematic curricular design helps university students from diverse disciplinary backgrounds develop a comprehensive understanding of AI technologies and ethics (Kong et al., 2021).

Taken together, existing studies demonstrate that AI literacy research is gradually forming a complete developmental chain, spanning theoretical framework construction, scale development, and pedagogical implementation across different educational stages. Nonetheless, several limitations remain. Current research has not fully revealed how various influencing factors interact in complex ways to dynamically shape and enhance AI literacy. In other words, while prior studies have identified which factors may matter, it remains unclear through what mechanisms and pathways these factors exert their influence. This lack of exploration into influence pathways makes it difficult for educators to implement precise interventions and prevents them from identifying the key leverage points that drive the endogenous development of AI literacy. Therefore, to bridge the gap between theory and practice, the present study draws on Conservation of Resources Theory to develop an integrated influence-path model, which serves as the theoretical framework guiding this research. Based on this model, the study further examines the generative mechanisms of AI literacy as a strategic resource, thereby deepening our understanding of AI literacy and enriching its related research field.

## 2 Hypotheses

### 2.1 External support

External support refers to the guidance, assistance, and contextual resources that individuals receive from teachers, peers, and family members during the process of learning and using AI-related tools, systems, or courses. This concept emphasizes that learners do not develop technical literacy in isolation; rather, they receive continuous help and feedback within social, organizational, and educational environments. Such support facilitates the acquisition of skills, comprehension of knowledge, and the development of ethical judgment. In the context of AI education, external support is particularly critical because AI technologies not only involve complex algorithms and interactive operations but also encompass interdisciplinary knowledge such as ethics, data security, and sustainability. Without adequate guidance or resource assurance, learners often struggle to fully understand and apply AI concepts.

Existing studies demonstrate that external support exerts a significant positive influence on the development of AI literacy. For example, research has shown that multi-agency collaboration and systematic educational support can markedly improve students’ AI understanding and practical skills (Perchik et al., 2023). Similarly, in the context of education for sustainable development, external support provided through teacher guidance, task scaffolding, and diverse learning resources has been found to significantly enhance students’ AI literacy, conceptual understanding, and sustainability-related judgment (Alamäki et al., 2024). These findings indicate that external support not only helps learners master technical operations but also enables them to connect AI applications with complex real-world problems, thereby fostering critical thinking.

Moreover, external support is reflected in curriculum design, learning tools, and feedback mechanisms. Systematic curricula, regular feedback, and personalized teacher guidance have been shown to improve students’ conceptual understanding, skill application, and ethical awareness (Kong et al., 2023). Likewise, even basic AI courses can effectively enhance students’ AI literacy and learning confidence under adequate teacher support, suggesting that the value of external support is consistent across educational levels (Chen and Zhang, 2025).

In K–12 education, external support is equally indispensable. Through guiding students in using conversational AI tools, explaining underlying model mechanisms, and designing interactive tasks, teachers play a crucial role in enhancing students’ understanding and application of AI (Van Brummelen et al., 2021). This illustrates that teachers not only provide technical assistance but also support the development of cognitive and ethical understanding.

Within library and academic resource environments, external support is embodied through course frameworks and resource-oriented learning environments. Structured curricula and abundant digital resources offer essential scaffolding for self-directed learning, significantly improving students’ mastery of AI technologies and information literacy (Chigwada, 2024). Additionally, studies indicate that in blended learning environments, continuous support from teachers and digital platforms enables learners to receive effective guidance in both self-regulated and collaborative learning, thereby promoting technological understanding and practical capabilities (Fathahillah et al., 2023).

Taken together, external support encompasses not only teacher guidance, peer collaboration, and educational resources but also curriculum design, feedback mechanisms, and environmental assurance. These forms of support reduce learning difficulty, increase task success rates, and strengthen learners’ understanding and confidence in AI technologies, thereby significantly promoting the development of AI literacy.

*Hypothesis 1 (H1)*: External support has a significant positive effect on AI literacy.

A large body of research shows that external support can significantly enhance teachers’ confidence and beliefs regarding the use of AI technologies. Supportive conditions in educational contexts—including resources, technical guidance, and organizational culture—substantially increase teachers’ self-efficacy in AI integration (Oran, 2023). When teachers receive sufficient training and systematic support, their AI-TPACK competence improves, thereby strengthening their AI instructional self-efficacy (Du et al., 2025).

In subject-teaching contexts, external support helps learners better understand the role of AI in the classroom, enhancing both their integration competence and their self-efficacy (Yehya et al., 2025). Furthermore, when learners receive continuous training and external assistance, their AI teaching self-efficacy increases significantly (Lu et al., 2024; Yang et al., 2024).

*Hypothesis 2 (H2)*: External support has a significant positive effect on AI self-efficacy.

AI anxiety refers to feelings of worry, unease, and uncertainty experienced by teachers when facing AI technology integration. Research suggests that while appropriate external support may reduce stress associated with technology use, it may also increase anxiety in some situations due to intensified training requirements or elevated performance expectations. Existing literature provides multi-level evidence to explain this complex relationship. For instance, during AI training programs, individuals who receive extensive technical guidance, curricular support, and resource provision may perceive increased pressure when learning new technologies, thereby elevating short-term AI learning anxiety (Hsu et al., 2023). Similarly, when external expectations rise—such as heightened instructional demands or pressure to integrate new technologies—teachers may experience greater burden, leading to increased anxiety (Hutchinson and Janiszewski Goodin, 2013).

Student-based research also reveals consistent patterns. External academic pressure can heighten anxiety (Killu et al., 2016), while task demands or the need to use new technologies may increase learners’ anxiety levels (Azher et al., 2010; Cooper et al., 2018). In AI education contexts, external support often entails new learning resources, new technical requirements, or new instructional objectives. These elements, as forms of “additional external inputs,” may heighten teachers’ perceptions of insufficient mastery, triggering anxiety responses. Thus, some studies argue that external support does not simply reduce anxiety; in fact, increases in information input, performance expectations, and training load may temporarily elevate AI-related anxiety.

*Hypothesis 3 (H3)*: External support has a significant positive effect on AI anxiety.

### 2.2 AI self-efficacy

AI self-efficacy refers to individuals’ belief in their ability to understand the functions of AI tools, applications, or systems, successfully complete AI-related tasks, and resolve operational problems. As AI technologies continue to grow in complexity, learners must engage with new forms of technical challenges—including algorithmic reasoning, model prediction, automated processes, and human–AI interaction. In this context, the level of AI self-efficacy becomes a crucial determinant of the depth of learners’ engagement. Existing studies have clearly demonstrated a close relationship between AI self-efficacy and AI literacy. AI self-efficacy is an important psychological factor predicting individuals’ ability to understand, use, and critically evaluate AI systems, including their ethical implications (Lim, 2023). Learners with higher AI self-efficacy are more willing to actively explore AI tools, and they tend to persist even in cognitively demanding tasks, thereby achieving higher-level technical competence and critical understanding.

Research from multiple educational levels further strengthens this association. For instance, studies employing structural equation modeling have shown that self-efficacy not only directly influences AI literacy but also indirectly enhances learners’ AI-related behavioral competence by strengthening their learning motivation (Fitriana, 2025). Similarly, AI self-efficacy has been identified as a significant mediating factor in AI learning processes, functioning as a core psychological mechanism that transforms learners’ interest into actual skills (Kaltenegger, 2024). Among adolescents, the importance of self-efficacy remains prominent. In addition, AI self-efficacy plays an essential role among educational leaders and teachers. For example, school principals’ AI self-efficacy significantly increases their willingness to adopt and apply AI technologies, further influencing school-level AI implementation and educational innovation (Bellibaş et al., 2025).

Overall, AI self-efficacy not only predicts learners’ ability to understand and operate AI systems but also shapes their learning motivation, usage intention, and ethical judgment. It forms a critical psychological foundation for the development of AI literacy. Therefore, AI self-efficacy should be considered a core component when building models of AI literacy.

Based on the literature above, this study proposes the following hypothesis:

*Hypothesis 4 (H4)*: AI self-efficacy has a significant positive effect on AI literacy.

### 2.3 AI anxiety

AI anxiety refers to individuals’ feelings of worry, unease, or perceived threat when encountering or using artificial intelligence technologies. Existing studies indicate that AI anxiety not only shapes users’ cognition and attitudes toward AI but also influences the development of their technological competencies (Cengiz and Peker, 2025). Research has shown that when students feel anxious about AI, they tend to devote more attention to the potential risks and capability boundaries of AI systems. This heightened attention further strengthens their evaluation and decision-making processes regarding technology, thereby contributing to the enhancement of their AI literacy.

Although anxiety is generally regarded as a negative emotional state, some studies have found that when anxiety coexists with technological awareness, users often attempt to reduce uncertainty by increasing their attention to technology and are more inclined to actively use AI tools to improve efficiency or compensate for perceived shortcomings (Liu et al., 2025). Taken together, AI anxiety may inhibit technology use; however, under certain conditions, it may also stimulate users’ motivational drive, making them more willing to actively explore or continue using AI tools.

Based on the above literature, this study proposes the following hypothesis:

*Hypothesis 5 (H5)*: AI anxiety has a significant positive effect on AI literacy.

## 3 Methods

### 3.1 Participants

The study involved 405 participants aged between 17 and 23 years. Females constituted the majority, with 238 individuals representing 59% of the total sample. Ethical approval was obtained from the institutional ethics committee affiliated with the first author, in accordance with APA ethical guidelines. This study adopted a combination of convenience sampling and snowball sampling to recruit participants, collecting data through an online questionnaire platform. The target population was full-time undergraduate students enrolled in higher education institutions. Larger samples (e.g., 300–500) are preferred for models with multiple latent constructs and pathways to ensure stable parameter estimates and sufficient statistical power (Kline, 2016.). This study received 405 questionnaires, with a total number exceeding 300. Therefore, it can meet the data volume requirements of the structural equation model (SEM). The sample was designed to cover students from different academic disciplines (e.g., science and engineering, humanities and social sciences, business and management) and various grade levels to enhance the generalizability of the results.

### 3.2 Instruments

All constructs were measured using a five-point Likert scale (1 = “strongly disagree,” 5 = “strongly agree”). Each scale was adapted from validated instruments to fit the context of this study, and reliability and validity assessments were conducted accordingly. The measurement instruments are described as follows:

AI Self-Efficacy Scale: This study adapted the General Self-Efficacy Scale for AI (GSE-6AI), which was developed for university students to assess their confidence in achieving goals with the assistance of artificial intelligence technologies (Morales-García et al., 2024). A representative item is: “With the assistance of AI, I can find ways to overcome obstacles and achieve my goals.” In this study, the scale demonstrated good internal consistency, with a Cronbach’s *α* of 0.84.

External Support Scale: The measure of external support was adapted from research on academic support (Chen, 2005), incorporating support from parents, teachers, and peers. Items covered dimensions such as social monitoring, direct assistance, and relational communication. A sample item is: “My parents help me find ways to solve academic problems.”

AI Anxiety Scale: AI anxiety was measured using the AI Anxiety Scale (Wang and Wang, 2022), which assesses students’ worry and tension related to artificial intelligence technologies. The original instrument consists of 21 items across four factors, including “learning anxiety” and “job replacement anxiety,” and has demonstrated strong psychometric properties. Contextual adaptations were made for the purposes of this study.

AI Literacy Scale: AI literacy was assessed using a scale adapted from Wang et al. (2023), which evaluates students’ AI literacy across dimensions such as awareness, usage, evaluation, and ethics. A representative item is: “I can identify the AI technologies used in the products and applications I interact with.”

### 3.3 Procedure

A pilot test was first conducted to evaluate item quality and assess reliability and validity using Cronbach’s *α*. Following the pilot, confirmatory factor analysis (CFA) was performed in AMOS 29 to examine the convergent and discriminant validity of the measurement model. Structural equation modeling (SEM) was then applied using the maximum likelihood estimation method to test the hypothesized relationships among external support, AI self-efficacy, AI anxiety, and AI literacy. Model fit was evaluated with multiple indices, including *χ*^2^/df, RMSEA, CFI, TLI, and SRMR.

### 3.4 Data analysis

This study adopted an explanatory sequential mixed-methods design, in which quantitative data were first collected and analyzed to identify general patterns, followed by qualitative interviews to further explain and enrich the quantitative findings. Quantitative survey data were analyzed using SEM in AMOS 29 to test the hypothesized structural relationships. To complement these findings, semi-structured interviews were conducted with 15 participants. Transcripts were imported into NVivo 15 for thematic analysis, following an iterative process: open coding to identify initial concepts, axial coding to explore relationships between categories, and selective coding to refine overarching themes. This approach enabled the integration of statistical patterns with participants’ perspectives, capturing both cognitive and affective dimensions of AI literacy.

## 4 Results

### 4.1 Confirmatory factor analysis results: factor loadings and reliability indicators

To evaluate the quality of the measurement model, this study conducted confirmatory factor analysis (CFA) on all core constructs to assess their reliability and convergent validity. The results are presented in Table 1. The evaluation focused on several key indicators: standardized factor loadings, which assess the extent to which each observed item represents its latent construct (values above 0.70 are considered ideal, and values above 0.50 acceptable); and composite reliability (CR), which indicates the internal consistency among items measuring the same construct, with values above 0.70 demonstrating satisfactory reliability.

**Table 1:** Confirmatory factor analysis results: factor loadings and reliability indicators.

Construct	Items	Unstd.	S.E.	Z-value	*p*	Std.	CR
External_Support	ES1	1				0.914	0.862
	ES2	0.884	0.073	12.078	***	0.821	
	ES3	0.112	0.06	1.855	***	0.724	
AI_Anxiety	AA1	1				0.768	0.864
	AA2	1.001	0.072	13.827	***	0.792	
	AA3	1.160	0.077	15.003	***	0.909	
AI_Self_Efficacy	ASE1	1				0.581	0.781
	ASE2	1.235	0.144	8.55	***	0.682	
	ASE3	1.046	0.13	8.019	***	0.753	
	ASE4	1.266	0.15	8.436	***	0.726	
AI_Literacy	AL1	1				0.774	0.813
	AL2	0.753	0.124	3.428	***	0.624	
	AL3	0.682	0.146	4.563	***	0.632	
	AL4	1.023	0.164	6.228	***	0.845	

***p < 0.001.

The standardized factor loadings for items ES1 (0.914), ES2 (0.821), and ES3 (0.724) were all highly significant at the *p* < 0.001 level. This indicates that the items strongly represent the construct. The composite reliability for this construct was 0.862.

The measurement model for the AI Anxiety construct demonstrated excellent performance. All three items (AA1: 0.768, AA2: 0.792, AA3: 0.909) showed standardized factor loadings, each statistically significant at *p* < 0.001. The high composite reliability of 0.864 further confirms that the scale exhibits strong internal consistency and desirable convergent validity.

The AI Self-Efficacy construct also exhibited acceptable measurement quality. The four items had standardized loadings ranging from 0.581 to 0.753, with ASE2 (0.682), ASE3 (0.753), and ASE4 (0.726) all exceeding the 0.60 threshold and showing statistical significance. Although the loading for the first item (ASE1: 0.581) was relatively lower, the construct’s composite reliability reached 0.781, meeting the recommended threshold and indicating acceptable internal consistency. Although the AVE value for the AI Self-Efficacy construct is slightly below the recommended threshold, this issue is not uncommon for emerging constructs and does not necessarily indicate inadequate convergent validity when composite reliability exceeds 0.70. According to Fornell and Larcker (1981), constructs with sufficient composite reliability can still demonstrate acceptable convergent validity even when AVE falls marginally below the 0.50 threshold. In this study, all standardized factor loadings exceed 0.50, with most exceeding 0.60, indicating acceptable item-level convergence. Therefore, the AI Self-Efficacy construct was retained in the model due to its theoretical relevance and satisfactory overall reliability.

The measurement results for the AI Literacy construct fell within the acceptable range. The standardized loadings of its items varied from 0.624 to 0.845. Among them, AL1 (0.774) and AL4 (0.845) demonstrated strong loadings, while AL2 (0.624) and AL3 (0.632) fell below the ideal 0.70 benchmark but exceeded the acceptable threshold of 0.50. The composite reliability of this construct was 0.813, indicating adequate internal consistency across the items. Although AL2 and AL3 present relatively lower factor loadings compared with the commonly recommended 0.70 threshold, they were retained based on both statistical and theoretical considerations. From a statistical perspective, their loadings remain above the minimum acceptable level (0.50) (Hair et al., 2019), and their inclusion does not compromise the overall composite reliability or convergent validity of the construct. More importantly, from a content validity perspective, AL2 and AL3 capture conceptually essential dimensions of AI literacy (e.g., applied understanding and critical engagement with AI systems) that are particularly important in the context of this emerging construct, where theoretical operationalization is still evolving. Therefore, retaining these items ensures comprehensive construct coverage and avoids underrepresentation of key facets of AI literacy.

### 4.2 Discriminant validity

To assess whether the latent variables in the measurement model demonstrated adequate discriminant validity—ensuring that the constructs are statistically distinct from one another—the average variance extracted (AVE) and the square root of AVE for each construct were calculated and compared with the inter-construct correlations (see Table 2). Following the Fornell–Larcker criterion, a measurement model is considered to exhibit satisfactory discriminant validity when the square root of each construct’s AVE exceeds its correlations with any other construct.

**Table 2:** Average variance extracted.

Constructs	External_Support	AI_Anxiety	AI_Self_Efficacy	AI_Literacy
External_Support	**0.823**			
AI_Anxiety	0.404	**0.825**		
AI_Self_Efficacy	0.505	0.204	**0.688**	
AI_Literacy	0.455	0.507	0.581	**0.724**

The bold values on the diagonal represent the square roots of the AVE for each construct, while the off-diagonal values indicate the Pearson correlations between constructs.

As shown in Table 2, the square roots of the AVE for all constructs are greater than their correlations with other constructs, indicating good discriminant validity. For instance, the square root of the AVE for external support (0.823) is higher than its correlation with AI self-efficacy (0.505), and the square root of the AVE for AI literacy (0.724) exceeds its correlation with AI self-efficacy (0.581). These results demonstrate that the measurement model exhibits good discriminant validity, with the four core constructs being statistically distinct and separable. Except for AI self-efficacy, the AVE values of the remaining three latent variables (external support, AI anxiety, and AI literacy) all exceed the recommended threshold of 0.50, indicating that their measurement items effectively converge and exhibit good convergent validity.

### 4.3 Verification of structural equation model fit

**Figure 1:**
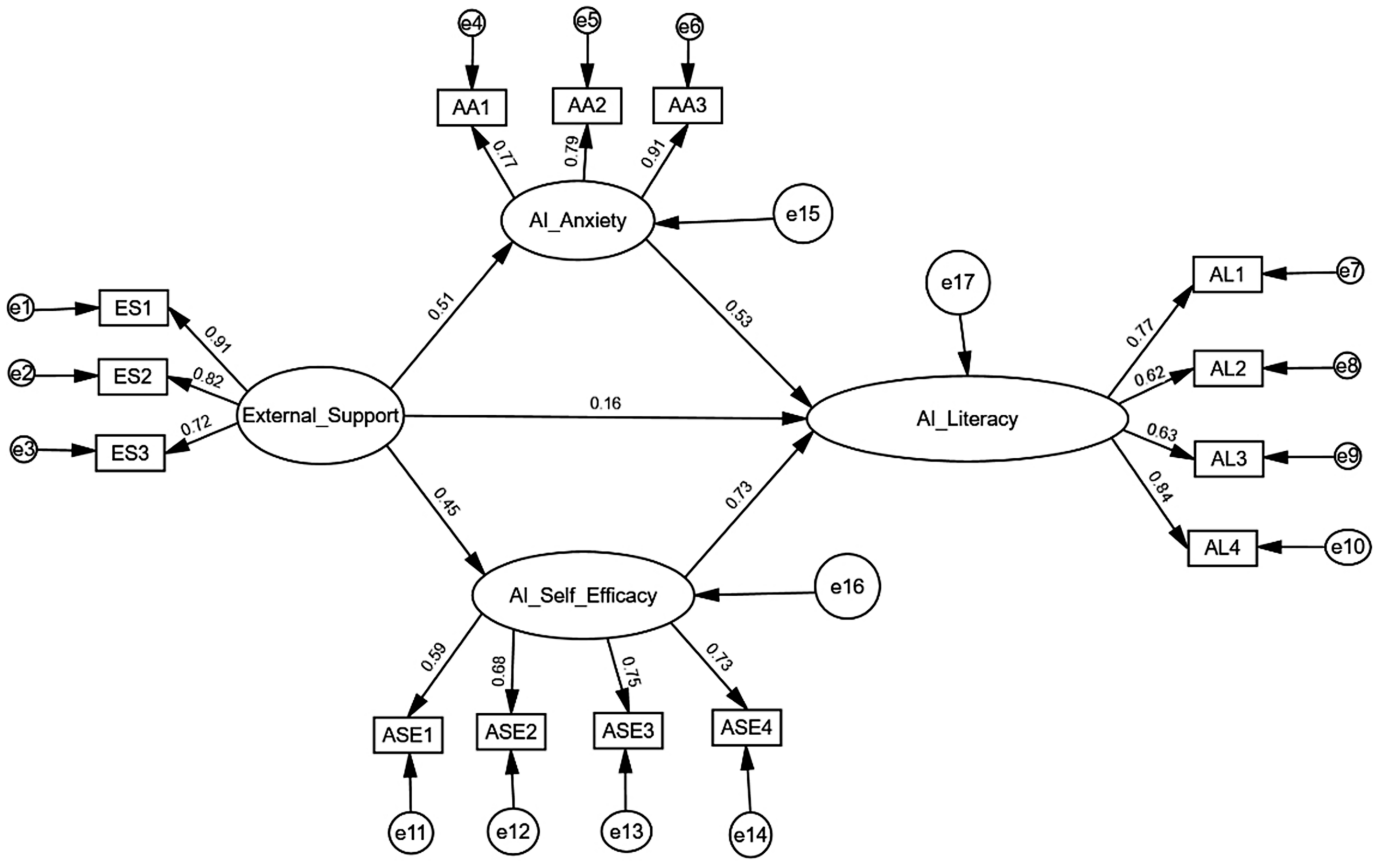
Structural equation model fit.

To test the proposed theoretical model and research hypotheses, structural equation modeling (SEM) was conducted following the assessment of the measurement model (see Figure 1). The model fit indices indicate a good fit between the theoretical model and the data: *χ*^2^/df = 2.15, CFI = 0.94, TLI = 0.92, RMSEA = 0.067, and SRMR = 0.045, with all indices meeting the widely accepted criteria for a good fit.

### 4.4 Path hypothesis testing

Based on the results presented in Table 3, all five hypotheses proposed in this study were statistically supported (*p* < 0.001). External support exhibited significant positive direct effects on AI anxiety (*β* = 0.505), AI self-efficacy (*β* = 0.454), and AI literacy (*β* = 0.163), confirming hypotheses H1, H2, and H3. Among all direct paths, the effect of AI self-efficacy on AI literacy was the strongest (*β* = 0.728), supporting H4. Additionally, AI anxiety demonstrated a significant positive direct effect on AI literacy (*β* = 0.527), supporting H5. To ensure consistency between the narrative and Table 3, all reported standardized path coefficients in the text are directly extracted from the final SEM output generated in AMOS 29, based on maximum likelihood estimation. The coefficients reported in Table 3 represent standardized regression weights after model convergence, and all values in the narrative have been cross-checked against the final model output to ensure full alignment.

**Table 3:** Summary of hypotheses testing results.

Regressed variables	Standardized estimate	SE	*p*	Decision
AI_Anxiety ← External_Support	0.505	0.042	***	Accept
AI_Literacy ← External_Support	0.163	0.034	***	Accept
AI_Self_Efficacy ← External_Support	0.454	0.043	***	Accept
AI_Literacy ← AI_Anxiety	0.527	0.071	***	Accept
AI_Literacy ← AI_Self_Efficacy	0.728	0.115	***	Accept

***p < 0.001.

### 4.5 Interviews with high anxiety—high literacy groups

The rapid integration of generative artificial intelligence into higher education has produced a paradoxical psychological profile: a cohort of students marked by both high technological literacy and elevated AI-induced anxiety. Traditional technology adoption models posit that increased literacy mitigates anxiety by enhancing confidence and perceived ease of use. However, emerging evidence suggests that advanced understanding of AI does not necessarily reduce fear; rather, it often redirects concern toward structural, ethical, and existential risks.

This qualitative supplement presents a thematic analysis of 15 purposively sampled students classified within the “High Anxiety–High Literacy” (HAHL) quadrant(Table 4). High literacy was defined as a mean score ≥4 on the AILS, and high anxiety as a score ≥4 on the AIAS. Data were collected through semi-structured interviews. Each interview was conducted individually via conferencing, lasting approximately 30 min. An interview protocol was developed to explore students’ experiences with AI use, their perceptions of anxiety, and their coping strategies. Data collection continued until thematic saturation was reached, defined as the point at which no new first-order codes or conceptual categories emerged across consecutive interviews. Specifically, after the 13th interview, preliminary coding indicated repetition of existing themes (e.g., delegation anxiety and surveillance anxiety), and the final two interviews were used to confirm theoretical stability rather than generate new categories. This suggests that thematic redundancy was achieved within the HAHL subgroup.

**Table 4:** Purposive sampling framework and participant selection criteria.

Code of participant	Grade	Field
S1	Freshman year	Engineering
S2	Freshman year	Social Science
S3	Freshman year	Social Science
S4	Sophomore year	Social Science
S5	Junior year	Engineering
S6	Sophomore year	Arts
S7	Freshman year	Arts
S8	Junior year	Social Science
S9	Junior year	Arts
S10	Sophomore year	Arts
S11	Junior year	Social Science
S12	Sophomore year	Arts
S13	Junior year	Engineering
S14	Sophomore year	Engineering
S15	Sophomore year	Engineering
S13	Junior year	Engineering

Through analysis of their lived experiences, we identify the mechanisms by which these students convert potentially debilitating fear into facilitative motivation, drawing on both internal coping strategies and external support systems.

The purposive sampling strategy utilized for this supplement targeted 15 students who met the rigorous criteria for the HAHL quadrant. These participants represent an “extreme case” in educational psychology, as their profile contradicts the traditional expectation that literacy leads to a linear reduction in anxiety. Instead, these students exhibit what some scholars describe as “vigilant trust”—a mode of engagement where openness to technology is accompanied by a sustained awareness of uncertainty and risk.

#### 4.5.1 Theme 1: the anatomy of high-literacy anxiety

For HAHL students, anxiety is not a generalized emotion but a context-sensitive, cognitively structured experience. Across grades and disciplines, participants expressed concerns rooted in their understanding of how AI reshapes intellectual labor, with four main dimensions: delegation anxiety, surveillance anxiety, creative displacement anxiety, and ethical vigilance.

Delegation anxiety was prominent among upper-year students. S5, a junior engineering student, reflected, “*When AI writes part of my code or summarizes research for me, it feels efficient—but I worry that I’m outsourcing the very struggle that trained me.*” S11, a junior social science student, noted, “*Research used to mean digging through arguments myself. If I rely on AI too much, I’m not sure I’ll maintain that depth.*”

Even freshmen showed early awareness of potential cognitive atrophy. S3, a first-year social science student, explained, “*I know AI can make things easier, but if I start depending on it now, I might never fully build my own analytical skills.*” Anxiety thus reflects a protective response against losing intellectual agency rather than incompetence.

Surveillance anxiety was more evident in ethically sensitive fields. S9, a junior arts student, observed, “*Every prompt leaves a trace. I’m aware that these systems collect more than we realize.*” S8, a junior social science student, noted, “*The data we input today could shape how algorithms treat people tomorrow.*” Engineering students also voiced structural concerns. S13 remarked, “*These models are trained on massive datasets. That makes me cautious about what I share.*”

Across years and disciplines, heightened literacy amplified awareness of data governance and algorithmic bias. Students feared long-term implications of AI embedding rather than operational failure. High-literacy anxiety is thus reflective, anticipatory, and discipline-sensitive, shaped by academic maturity and epistemic positioning.

#### 4.5.2 Theme 2: transforming debilitative fear into facilitative motivation

A key finding is not just the presence of anxiety, but its transformation. While debilitative anxiety causes hesitation or avoidance, HAHL students often shift toward facilitative anxiety—an alert state that enhances engagement. This shift is driven by cognitive appraisal: viewing AI not as an uncontrollable threat, but as a challenge requiring preparation and strategy.

For some sophomores, this transition was linked to growing confidence. S14, a sophomore engineering student, reflected, “*I was anxious about being replaced, especially in technical tasks. But once I understood the system’s limitations, I realized it still needs human supervision. That made me focus more on strengthening my own fundamentals.*” S10, a sophomore arts student, noted, “*The fear did not disappear. It changed. Now it reminds me to think more carefully and not rely blindly on outputs.*”

Among upper-year students, the shift was more pronounced. S8, a junior social science student, explained, “*AI makes things faster, but it also raises the bar. Knowing that pushes me to be sharper, not passive.*” Anxiety becomes a signal of responsibility rather than paralysis.

This transformation is often accompanied by proactive innovation behavior. S12, a sophomore arts student, stated, “*I’ll use AI to brainstorm, but I always rewrite in my own structure. That’s how I make sure the thinking is still mine.*” S15, a sophomore engineering student, added, “*If I use AI for debugging, I make myself explain the logic afterward. Otherwise I feel like I did not really learn it.*”

These accounts show calibrated engagement rather than avoidance or overreliance. Across disciplines and years, facilitative anxiety emerges not as absence of fear, but as fear reframed and mobilized toward competence.

#### 4.5.3 Theme 3: coping through resource accumulation (COR theory)

Consistent with Conservation of Resources (COR) theory, HAHL students framed AI literacy not merely as a technical skill, but as a strategic resource protecting them from potential losses in academic standing, professional relevance, or employability. Literacy thus functions as a conditional asset, reducing vulnerability in an AI-mediated environment.

Across disciplines, students saw competence as security. S2, a freshman in social science, reflected, “*If I understand how AI works, I feel less likely to be replaced by it. Ignorance would make me more anxious.*” S6, a sophomore arts student, added, “*Knowing the system’s strengths and weaknesses gives me a sense of control. It feels like having armor.*”

Digital competence also fostered digital resilience. When faced with technical errors or ambiguous AI outputs, high-literacy students remained composed. S4, a sophomore social science student, noted, “*Sometimes the output is wrong or biased. But instead of panicking, I treat it as something to debug.*” S15, a sophomore engineering student, stated, “*If it fails, I just adjust the prompt or cross-check. It does not feel like a crisis.*”

An unexpected pattern was exploratory vigilance resembling morbid curiosity. S7, a freshman arts student, shared, “*I sometimes ask it controversial questions just to see where it breaks.*” S14, a sophomore engineering student, remarked, “*Testing its biases helps me understand what it cannot be trusted with.*” Such behaviors function as uncertainty-reduction strategies, transforming diffuse anxiety into targeted vigilance. Resource accumulation thus does not eliminate anxiety but reorganizes it into a structured, manageable response.

#### 4.5.4 Theme 4: professional identity and the future outlook

For students in professionally oriented disciplines, AI literacy is closely linked to emerging career identity. Recognizing that AI can perform tasks traditionally associated with expert authority often triggers professional achievement anxiety. Rather than fearing direct replacement, students question how expertise itself is being redefined.

This concern was especially evident among those preparing for high-responsibility roles. S6, a sophomore in arts planning to enter education, reflected, “*If AI can generate lesson plans or explain concepts instantly, what makes a teacher valuable?*” Similarly, S9, a junior arts student, noted, “*It’s unsettling to think that knowledge delivery is no longer the main differentiator.*” Their anxiety centers less on competence than on professional distinctiveness.

Within the HAHL group, however, uncertainty frequently led to re-conceptualization. S12 explained, “*Maybe the role shifts. Instead of competing with AI, I need to know how to guide it and interpret it.*” S8 likewise observed, “*AI can process information, but it does not understand context the way humans do. That’s where I see my future value.*” Students increasingly framed themselves as coordinators of hybrid intelligence, responsible for oversight and ethical judgment.

Motivation mediates this shift. When uniquely human capacities—such as empathy and contextual reasoning—are seen as complemented by AI, engagement rises. As S2 stated, “*If AI handles routine tasks, I can focus more on understanding people.*” S5 added, “*It pushes me to develop the parts AI cannot replicate.*”

Overall, literacy alone does not sustain engagement; it is the perceived relevance of human contribution that transforms anxiety into forward-looking motivation.

## 5 Discussion

### 5.1 The relationship between external support and AI literacy

The path analysis results of this study indicate that external support has a significant positive effect on university students’ AI literacy. The findings suggest that external support serves as a crucial driver for students’ sustained engagement with AI learning and the deepening of their understanding. From the perspective of external resource allocation, systematic resource integration, instructional design, and supportive infrastructure are likely to be associated with learners’ AI competence development (Papagiannidis et al., 2021). External support encompasses not only hardware and technological resources but also course structures, teaching mechanisms, and organizational culture, collectively forming essential preconditions for the development of AI literacy.

The importance of external support is further corroborated in instructional models and blended learning environments. Research has shown that teacher guidance, platform support, and structured learning tasks are associated with improved students’ digital competencies and AI-related skills (Wang and Rao, 2024). Similarly, under blended learning conditions, continuous teacher feedback and access to platform resources may facilitate students’ understanding of AI operations and underlying principles (Fathahillah et al., 2023). Moreover, studies in cross-cultural and collaborative learning environments emphasize the role of external support in promoting AI literacy. Cross-cultural collaboration, peer support, and contextualized tasks encourage learners to engage actively in hands-on practice and critical thinking, thereby fostering the development of higher-order AI literacy (Korte et al., 2024). The results of this study suggest that peer collaboration and teacher support also play key roles in students’ AI literacy development. Support for information resources, guidance on learning strategies, and training in digital tool usage have a significant synergistic effect on enhancing both information literacy and AI literacy (Namdas, 2024).

The development of AI literacy is not solely dependent on conceptual learning; it highly relies on the richness of resources and the completeness of learning support systems. The positive effect of external support on AI literacy is further validated by research emphasizing that diversified learning resources, teacher guidance, and contextualized tasks may contribute to students’ AI understanding, ethical judgment, and practical skills (Alamäki et al., 2024). The impact of external support extends beyond learning resources or teacher guidance, functioning as an integral component of the “learning ecosystem.” It enables students to transform preliminary AI knowledge into practical skills and critical understanding (Zuo et al., 2025). Therefore, when promoting AI education reform in higher education, the focus should be on establishing a stable, sustainable, and adaptive supportive teaching ecosystem rather than merely providing content or tools. The formation of AI literacy is, in essence, an “ecological construction” that requires multidimensional synergy in resources, instruction, feedback, and culture, thereby fostering learners’ comprehensive understanding and competence development.

### 5.2 The relationship between external support and AI self-efficacy

External support has a significant positive effect on university students’ AI self-efficacy. This finding suggests that external support serves as an important environmental factor in shaping learners’ beliefs about their capabilities. In AI-supported learning environments, learners’ perceptions of external support—such as teacher guidance, learning resources, and technological conditions—are associated with their sense of task controllability and confidence (Qiu, 2025). When students receive adequate technical assistance, clear learning structures, and emotional support while using AI tools, their self-efficacy is likely to be enhanced. From the perspective of intervention and competence development, external support may influence self-efficacy by improving learners’ operational proficiency and accumulation of successful experiences. AI tools and assistive systems that provide immediate feedback, clear procedural guidance, and personalized instruction can help students gain a stronger sense of mastery over technology, thereby being associated with higher AI self-efficacy (Parsakia, 2023). Structured learning support and resource provision enable students to continually accumulate successful experiences in practice, which has been widely recognized as a core pathway for increasing self-efficacy. When learners receive sufficient course resources, teacher care, and peer support, their self-efficacy levels tend to be higher (Van Dinther et al., 2011). Enhancing task controllability through structured external support is a key mechanism for supporting learners’ self-efficacy (Walker, 2003; Zakariya, 2022).

The facilitative effect of external support is not limited to specific domains, such as mathematics or reading and writing, but broadly applies to the emerging and complex field of AI learning. Structured technical training, timely teacher feedback, visualized learning materials, and peer collaboration can reduce learners’ perceived task complexity, allowing them to gain a stronger sense of control and higher expectations of success when using AI technologies, thereby effectively enhancing AI self-efficacy. External support can also indirectly promote learners’ self-efficacy by alleviating learning-related anxiety and enhancing emotional security (Cattelino et al., 2019). From resource provision to instructional support, and from emotional regulation to task structuring, external support creates a favorable environment that strengthens learners’ confidence and expectations of success. Future AI education reforms should continue to improve supportive teaching ecosystems, providing multidimensional support through curriculum design, technological platforms, feedback mechanisms, and collaborative networks, thereby enhancing students’ AI self-efficacy and practical competence.

### 5.3 The relationship between external support and AI anxiety

The results of this study indicate a significant positive relationship between external support and AI anxiety. That is, the stronger the external support, the higher the students’ AI anxiety. This finding does not entirely align with the traditional view that “support reduces anxiety,” but it is highly consistent with some studies highlighting the stress effects associated with technology-based learning.

Research has shown that when learners receive more intensive guidance, supervision, or resource input, they may experience additional emotional stress due to the increased technological demands (Teng, 2024). Structured external learning support can sometimes raise the complexity of learning tasks, exposing learners more frequently to technical details and performance evaluations, thereby potentially triggering stronger anxiety (Bellini et al., 2016). In high-demand technological learning environments, external support is often accompanied by higher expectations, stricter task requirements, and more frequent feedback mechanisms, which students may interpret as “pressure inputs” (Pesántez-Avilés et al., 2024). Therefore, external support is not only a source of resources and assistance but also a symbol of “performance demands.” As resources increase, technology becomes more advanced, and tasks become more intensive, the cognitive effort, time investment, and performance responsibility required from learners also rise, leading to a short-term increase in AI anxiety.

In other words, the role of external support in AI learning is “double-edged.” While it provides conditions and guarantees for learning, it can also inadvertently intensify learners’ task-related stress and self-evaluation burden, resulting in elevated anxiety levels. When constructing AI education support systems, universities should avoid the structural effect of “support as burden” by adjusting task difficulty, reducing evaluative pressure, and enhancing psychological support, thereby ensuring that external support effectively does not necessarily reduce but may also relate to changes in anxiety.

### 5.4 The relationship between AI self-efficacy and AI literacy

AI self-efficacy has a significant positive impact on artificial intelligence literacy. Self-efficacy is a core psychological mechanism that is believed to drive individuals to continuously explore, deepen understanding, and develop practical skills in the field of AI technology (Olivier et al., 2019). As a belief in one’s capabilities, self-efficacy is one of the most stable and important predictors of learning behavior, exerting profound influence on learning engagement, persistence, and overall learning outcomes (Al-Abyadh and Abdel Azeem, 2022).

In AI learning contexts, self-efficacy not only affects learners’ behavioral motivation but also shapes the depth of their engagement and persistence when confronting complex tools and uncertain technological tasks. Students with high self-efficacy are more willing to actively experiment with new AI tools, fine-tune models, or explore different application scenarios, thereby accumulating technical experience and gaining a deeper understanding of knowledge structures (Kong et al., 2025). Self-efficacy promotes the development of overall AI literacy by enhancing learning engagement and enabling learners to make more mature judgments and reasoning across various contexts (He et al., 2025).

Moreover, AI self-efficacy is closely linked to learners’ creative identity and technological mindset. Learners with high self-efficacy are more likely to cultivate a positive attitude toward technology, which further expands their ability to apply AI in complex scenarios (Li et al., 2025). AI self-efficacy serves as a key driver for enhancing artificial intelligence literacy. High self-efficacy not only strengthens students’ interest and confidence in AI technology but also fosters the development of advanced literacy, including operational skills, analytical thinking, judgment, and ethical reasoning. Therefore, in the construction of AI education systems, fostering self-efficacy should be regarded as a critical pathway to improving AI literacy, rather than merely an auxiliary factor.

### 5.5 The relationship between AI anxiety and AI literacy

There is a significant positive relationship between AI anxiety and artificial intelligence literacy, meaning that higher levels of AI anxiety tend to co-occur with higher AI literacy among learners. This finding differs from a large body of research on the relationship between anxiety and learning ability. Previous studies have indicated that anxiety can inhibit learners’ cognitive engagement, reduce persistence in complex tasks, and weaken the effectiveness of learning strategies (Cooper and Robinson, 1991). In high-demand technological learning environments, anxiety has been reported to be associated with reduced digital tools (Fathali et al., 2024). Similarly, higher AI anxiety is associated with lower digital and AI literacy among pre-service teachers, demonstrating a clear hindering effect (Ayduğ and Altınpulluk, 2025).

Some studies suggest that moderate anxiety may have a facilitative effect in certain learning contexts. Research indicates that anxiety is not entirely negative; under specific conditions, it can act as an activating factor that stimulates motivation and increases learning engagement. For instance, moderate language anxiety may enhance learners’ motivation, leading them to invest greater effort in tasks (Luo et al., 2020). Similarly, studies have found a positive relationship between test anxiety and study effort, where students with slightly higher anxiety levels are more willing to dedicate additional time to preparing for learning tasks (Heckel et al., 2021). Within a certain range, anxiety can improve attention sensitivity and task alertness, thereby promoting academic performance (Rani, 2025). Moderate anxiety can strengthen learners’ focus on learning tasks, resulting in more active engagement both in classroom and digital learning environments (Welesilassie and Nikolov, 2024).

### 5.6 Synthesizing the HAHL narrative

The transformation of AI anxiety into literacy-driven engagement is not a passive process of “getting used to the tool.” For the high-anxiety, high-literacy student, it is an active, often strenuous, cognitive and emotional endeavor. The thematic analysis demonstrates that these students leverage their advanced knowledge to engage in “targeted vigilance,” “proactive innovation,” and “resource accumulation.” They are not merely “adopters” of technology; they are “critical allies” who use their literacy as a shield against the technology’s potential harms. The future of higher education in the AI era depends on the ability of institutions to support this transformation. By shifting focus from technical “how-to” training to “critical agency” and “ethical competence,” universities can help students harness their anxiety as a facilitative force. The “Critical Alliance” framework, supported by robust instructor interaction and institutional guardrails, provides a viable path for students to remain autonomous, ethical, and mentally healthy in an increasingly automated world. Ultimately, the data from these 15 students suggests that the most successful learners are not those without fear, but those whose literacy is so profound that they know exactly what to fear—and how to build the resilience to face it. The “psychological black box” of AI adoption is thus opened through a combination of digital competence, interpersonal support, and the preservation of human cognitive agency.

### 5.7 Integration of quantitative and qualitative findings

The integration of quantitative and qualitative findings provides a more comprehensive explanation of how AI literacy, AI anxiety, and external support interact within the theoretical framework of this study. Grounded in COR Theory and complemented by Social Cognitive Theory, the results suggest that learners actively manage cognitive, emotional, and environmental resources when engaging with AI-supported learning systems. Quantitatively, external support was found to significantly enhance AI literacy and AI self-efficacy, while simultaneously increasing AI anxiety. These seemingly contradictory effects can be interpreted through COR theory, which posits that resource acquisition often co-occurs with heightened sensitivity to potential resource loss. In this context, external support functions not only as a facilitating resource but also as a visibility amplifier of technological demands, thereby increasing learners’ awareness of uncertainty and potential risks. The qualitative findings further elaborate this mechanism by showing that students with high AI literacy and high anxiety do not experience anxiety as purely inhibitive, but rather transform it into “facilitative vigilance” through resource accumulation, strategic engagement, and reflective use of AI tools. This aligns with the quantitative finding that both AI anxiety and AI self-efficacy positively predict AI literacy, suggesting that anxiety can operate as a motivational signal when sufficient cognitive and external resources are available. From the perspective of Social Cognitive Theory, these results indicate a triadic reciprocal interaction among personal factors (AI literacy and self-efficacy), behavioral engagement (strategic AI use), and environmental inputs (external support). The interview data provide concrete behavioral evidence of this mechanism, showing how learners regulate their behavior through continuous feedback loops with AI systems and educational contexts.

Taken together, the quantitative and qualitative results converge to demonstrate that AI literacy development is not a linear outcome of reduced anxiety or increased support alone, but rather a dynamic resource-regulation process. Within this process, external support provides structural opportunities, self-efficacy enables sustained engagement, and anxiety can enhance vigilance and adaptive learning.

## 6 Limitations

This study, employing structural equation modeling, uncovered the complex relationships among external support, AI self-efficacy, AI anxiety, and AI literacy among university students. It must be acknowledged that there are some limitations in this study. First, the study relied on a cross-sectional design, collecting all data at a single time point. While SEM enables examination of pathways among variables, the findings remain correlational and cannot definitively establish causality or temporal dynamics. Future research could adopt longitudinal designs or experimental interventions to more rigorously test causal mechanisms and the evolution of AI literacy over time. Although this approach captured diversity in majors and year levels, the sample may not fully represent the broader population of Chinese university students, including those in vocational colleges or underrepresented regions. Therefore, the generalizability of the findings requires further validation across different educational stages, cultural contexts, and disciplinary groups. While this study incorporated both quantitative SEM analysis and qualitative insights from the High Anxiety–High Literacy cohort, the qualitative sample was limited in size and scope. Future studies could expand qualitative exploration to examine nuanced experiences across diverse student populations, enhancing the depth and transferability of findings.

## 7 Conclusion

Grounded in a structural equation model integrating external support, AI self-efficacy, AI anxiety, and AI literacy, this study systematically explored the mechanisms driving university students’ AI literacy development. The results indicate that all proposed hypotheses were statistically supported, revealing that AI literacy is shaped not only by environmental support and individual self-efficacy but also by the nuanced role of anxiety in facilitating engagement and critical awareness. Notably, high AI literacy does not eliminate anxiety; rather, it transforms concern into a proactive, reflective, and discipline-sensitive engagement. Students leverage internal coping strategies and external support to convert potential debilitative fear into facilitative motivation, highlighting the interplay of cognitive, emotional, and environmental factors in AI literacy development. These findings underscore the importance of integrated interventions that simultaneously enhance support structures, self-efficacy, and strategic engagement with AI tools, guiding both curriculum design and educational policy in AI-mediated learning environments.

## Data Availability

The raw data supporting the conclusions of this article will be made available by the authors, without undue reservation.
